# Uptake of hepatitis B vaccination and its determinants among health care workers in a tertiary health facility in Enugu, South-East, Nigeria

**DOI:** 10.1186/s12879-018-3191-9

**Published:** 2018-06-28

**Authors:** I. B. Omotowo, I. A. Meka, U. N. Ijoma, V. E. Okoli, O. Obienu, T. Nwagha, A. C. Ndu, D. O. Onodugo, L. C. Onyekonwu, E. O. Ugwu

**Affiliations:** 1 Department of Community Medicine, College of Medicine, University of Nigeria, Enugu Campus, Nigeria; 2 Department of Medicine, College of Medicine, University of Nigeria, Enugu Campus, Nigeria; 3 Department of Chemical Pathology, College of Medicine, University of Nigeria, Enugu Campus, Nigeria; 4 Department of Haematology, College of Medicine, University of Nigeria, Enugu Campus, Nigeria; 5 Department of Dermatology, College of Medicine, University of Nigeria, Enugu Campus, Nigeria; 6 Department of Obstetrics and Gynaecology, College of Medicine , University of Nigeria, Enugu Campus, Nigeria

**Keywords:** Health care workers, Hepatitis B, Hepatitis B vaccine, Nigeria

## Abstract

**Background:**

Hepatitis B vaccination is the most effective method of prevention for hepatitis B virus infection. It is a major public health problem in Nigeria, and health workers are at increased risk. This study determined the uptake of hepatitis B vaccination and assessed its determinants among health care workers (HCWs).

**Methods:**

A hospital-based cross-sectional study was conducted between July and August, 2016 using self-administered structured questionnaires among 3132 HCWs in University of Nigeria Teaching Hospital, Enugu, South-East, Nigeria. Data was analysed using SPSS version 22. Binary logistic regression analysis was used to identify factors that influenced uptake of vaccination. Ethical clearance was obtained from the Research Ethics Committee of the health facility.

**Results:**

The uptake of hepatitis B vaccination was 14.2% (*n* = 445). The number of doses received were: 3 doses (218/3132, 48.9%), 2 doses (71/3132, 16.0%), and one dose (156/3132, 35.1%). The reasons for non-uptake of vaccination included: cost of vaccine 48 (10.8%), ‘did not believe they could be infected’ 28 (6.6%), long vaccination schedule, and lack of time 150 (35.1%). The Odds for uptake of hepatitis B vaccination were 22% lower among nurses compared to doctors (AOR = 0.78, 95% CI = 0.54–0.98, *P* = 0.037). It increased with increasing age (AOR = 1.30, 95% CI = 1.08–1.59, *P* <  0.001), increasing duration of work in the hospital (AOR = 1.19, 95% CI = 1.09–1.32, *P* = 0.032), and was about twice higher among those that had tertiary education than others that had less education (AOR = 1.96, 95 CI = 0.76–5.07, *P* = 0.164).

**Conclusions:**

The uptake of hepatitis B vaccination was low among HCWs in Enugu, Nigeria. Age, staff category, and duration of work in the hospital, were independently associated with hepatitis B vaccination. Provision of adequate hepatitis B surface antigen screening facilities and vaccination sites where the cost of vaccination is subsidized for all HCWs is recommended.

## Background

Hepatitis B is spread through contact with blood and body fluids of an infected person. It is a major public health problem in Nigeria and health care workers (HCWs) including general physicians, surgeons, dental surgeons, nurses and other medical staff. These HCWs are at increased risk of acquiring the disease due to occupational exposure to blood and body fluids [1, 2]. Hepatitis B is a life-threatening liver infection caused by Hepatitis B Virus (HBV). It can cause chronic and often fatal liver diseases, such as liver cirrhosis and cancer. Globally, about a billion individuals have been infected with HBV at some point in their lifetime and almost 350 million people are chronically infected with HBV, out of which more than a million die annually from its related causes [3]. Majority of the infected cases are living in sub-Saharan Africa [4].

World Health Organization (WHO) reported that about two million health care workers risk occupational exposure to HBV each year and vaccination coverage is low among them [5]. The Department of Health and Human Services, Centers for Disease Control and Prevention (CDC) reported in United States that “the risk of being infected is dependent on the prevalence of the HBV carriers and frequency of exposure of HCWs to blood and body fluids and the infectivity of the virus” [6]. Health care workers in Nigeria are particularly at a greater risk because Nigeria is a holoendemic area, with HBV carrier rate of 15–37% [7]. The risk of acquiring HBV in some cadres of HCWs is four times greater than that of the general population [8].

Hepatitis B vaccination is the mainstay of HBV prevention and has been reported to reduce the risk of acquiring the infection virtually to zero [9, 10]. It is recommended for exposed HCWs as part of the universal precautions policy for protection of HCWs [6]. HCWs who are HBV negative after screening should take hepatitis B vaccination, while those who are HBV positive should be treated. However, vaccination among HCWs remains a challenge for many countries [11]. Some studies reported that all HCWs including administrative staff in a hospital can receive the hepatitis B vaccine [11]. The mode of transmission of HBV in health care settings is most often by needle prick injuries and poor adherence to universal precautions [12]. The prevention of occupational hazards requires a thorough knowledge of the risks and practical measures to be taken, and the need for HCWs workers to familiarize themselves with universal work precautions [13]. It is estimated that about a million HCWs had cut and puncture injuries per year [14].

There is wide implementation of policy and uptake of the hepatitis B vaccine in some countries such as UK, USA, and Israel [10]. In 2016, Nigeria developed a national guideline for prevention, care and treatment of HBV and hepatitis C virus (HCV) infections, and vaccination of HCWs was included as one of the preventive methods in health care settings. However, to the best of the authors’ knowledge, there is no policy or implementation of any policies that makes uptake of Hepatitis B vaccination compulsory to all HCWs in Nigeria. This study was conducted to evaluate the uptake of hepatitis B vaccination and its determinants among HCWs in a tertiary health facility in South-East, Nigeria.

## Methods

### Study area

This study was conducted between July and August 2016 among HCWs in University of Nigeria Teaching Hospital (UNTH), Enugu, South-East Nigeria. The UNTH is a tertiary health facility, and the national cardiothoracic center of excellence. The facility attends to patients from all over Nigeria.

### Study design and sampling technique

This was a hospital-based cross-sectional study. It was conducted prior to the free HBV and HCV screening programmes organised by Roche Products Limited in collaboration with the hospital’s Management. To the best of the authors’ knowledge, it was the first free HBV and HCV screening programme ever organized for all the hospital’s HCWs.

### Data collection

Data was collected using pre-tested self-administered structured questionnaires designed to collect information on socio-demographic characteristics, knowledge of transmission and risk factors of HBV, HBV status, uptake of hepatitis B vaccination, doses of vaccine received, and reasons for non-uptake of the vaccine by the participants.

### Participants in the study

**Fig. 1:**
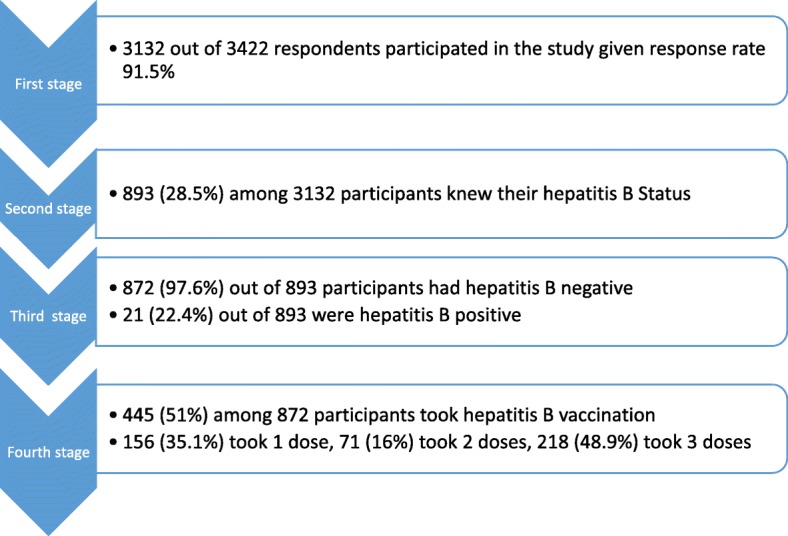
Participants flow chart

Figure 1 shows the respondents’ flow chart. The first stage showed that 3132 out of 3422 HCWs completed the questionnaires correctly given a response rate of 91.2%. In the second stage, 893 (28.5%) out of 3132 knew their HBV status. Third stage showed that 872 (97.6%) out of 893 participants that knew their HBV status were HBV negative, while 21 (2.4%) were HBV positive. The fourth stage showed the participants who have received hepatitis B vaccination and the doses received.

### Data analysis

Data collected were analysed using SPSS version 22 (SPSS Inc., Chicago, Illinois, USA). Descriptive analyses were expressed as percentages. Socio-demographic characteristics of the respondents (age, sex, marital status, level of education, and duration of work in the hospital) and how it affected the uptake of hepatitis B vaccination was determined. The relationship between the factors and uptake of hepatitis B vaccination was calculated using Chi-square test to determine significance at *p* <  0.05. Binary logistic regression analysis was performed to determine socio-demographic characteristics of respondents associated with uptake of hepatitis B vaccination and the number of doses received. Variables with statistical significance of *P* ≤ 0.2 in the bivariate models were included in the multivariate analysis. Strength of association was expressed using Odds ratio and statistical significance presented using *P*-values and 95% confidence intervals for odds ratio. For all analyses, P-values of < 0.05 were considered statistically significant.

### Operational definitions

Uptake of hepatitis B vaccination referred to respondents who have received at least one dose of the hepatitis B vaccine. HBV negative referred to absence of hepatitis B surface antigen (HBsAg), while presence of HBsAg was referred as HBV positive. ‘Knew their hepatitis B status’ referred to respondents who have been screened in the past and had prior knowledge of their hepatitis B status, whether positive or negative. Full hepatitis B vaccination referred to respondents who have received 3 doses of the vaccine.

### Ethical approval

Ethical approval was obtained from Health Research Ethics Committee of the tertiary health facility, and informed written consent was obtained from all the participants.

## Results

### Socio-demographic characteristics of the participants

The mean age of the participants was 39.4 ± 9.6 (range: 18–75) years. Majority 1151(36.7%) were in the 31–40 years age group. About three quarters 2237 (71.4%) had tertiary education, 2174 (69.4%) were married and 580 (18.5%) were nurses. Majority of the participants 1938 (61.9%) had worked for more than 5 years (Table 1).

**Table 1:** Socio-demographic characteristics of the respondents (*N* = 3132)

Socio-demographic characteristics	Doctors (*N* = 297)		Nurses/Pharmacists/Lab Workers (*N* = 716)		Admin staff (*N* = 2119)		Total *N* = 3132	
	*N*	%	*N*	%	*N*	%	*N*	%
Sex								
Male	185	62.3	277	38.7	489	23.1	951	30.4
Female	112	37.7	439	61.3	1630	76.9	2181	69.6
Age – group (Years)								
< 20	0	0	0	0	12	0.6	12	0.4
20–30	94	31.7	84	11.7	414	19.5	592	18.9
31–40	112	37.7	196	27.4	843	39.8	1151	36.7
41–50	66	22.2	298	41.6	558	26.3	922	29.5
51–60	21	7.1	138	19.3	273	12.9	432	13.8
> 60	4	1.3	0	0	19	0.9	23	0.7
Education Level								
Primary	0	0	0	0	269	12.7	269	8.6
Secondary	0	0	23	3.2	603	28.5	626	20.0
Tertiary	297	100	693	96.8	1247	58.8	2237	71.4
Marital status								
Married	198	66.7	510	71.2	1466	69.2	2174	69.4
Single	97	32.6	201	28.1	571	26.9	869	27.7
Separated	0	0	0	0	11	0.5	11	0.4
Widowed	2	0.7	5	0.7	71	3.4	78	2.5
Duration of work (Years of experience)								
< 1 Year	38	12.8	19	2.7	206	9.7	263	8.3
1–5 Years	152	51.2	101	14.1	678	32.0	931	29.8
> 5–10 Years	50	16.8	380	53.1	410	19.3	840	26.8
> 10 Years	57	19.2	216	30.1	825	39.0	1098	35.1

### Knowledge on transmission of hepatitis B infection

Table 2 shows that less than half of the participants knew that hepatitis B could be transmitted by needle prick, sharing of sharp objects, and sexual intercourse, while about 70% knew that blood transfusion was a mode of transmission. Only 3% believed it could be transmitted by shaking of hands and 16.3% did not know any mode of transmission.

**Table 2:** Knowledge on transmission of hepatitis B (*N* = 3132)

Transmitted by:	Doctors (*N* = 297)		Nurses/Pharmacists/Lab Workers (*N* = 716)		Admin staff (*N* = 2119)		Total *N* = 3132)	
	*N*	%	*N*	%	*N*	%	*N*	%
Shaking of hands								
Yes	11	3.7	24	3.4	49	2.3	84	2.7
No	286	96.3	692	96.6	2070	97.7	3048	97.3
Needle prick								
Yes	280	94.3	293	40.9	749	35.3	1322	42.2
No	17	5.7	423	59.1	1370	64.7	1810	57.8
Blood transfusion								
Yes	284	95.6	614	85.8	1282	60.5	2180	69.6
No	13	4.4	102	14.2	837	39.5	952	30.4
Heat								
Yes	12	4.0	57	8.0	140	6.6	209	6.7
No	285	96.0	659	92.0	1979	93.4	2923	93.3
Sexual intercourse								
Yes	249	83.8	524	73.2	759	0.4	1532	48.9
No	48	16.2	192	26.8	1360	99.6	1597	41.9
Drinking contaminated water								
Yes	49	16.5	105	14.7	232	10.9	386	12.3
No	248	83.5	611	85.3	1887	89.1	2746	87.7
Contamination from surfaces								
Yes	81	27.3	89	12.4	402	18.9	572	18.3
No	216	72.7	627	87.6	1717	81.1	2560	81.7
Sharing of sharp objects								
Yes	258	86.9	204	28.5	1027	48.5	1489	47.5
No	39	13.1	512	71.5	1092	51.5	1643	52.5
Don’t know								
Yes	0	0	26	3.6	486	22.9	512	16.3
No	297	100	690	96.4	1633	77.1	2620	83.7

### Participants in the study flow chart and their uptake of hepatitis B vaccination

Table 3 shows the participants in each stage of the study. A total of 297 (9.5%) doctors, 716 (22.9%) nurses/pharmacists/lab workers, and 2119 (67.7%) administrative staff were involved in the study. Participants who knew their HBsAg status were 893 (28.5%). Twenty one, 21 (2.4%) were HBsAg positive, while 872 (97.6%) were HBsAg negative. Among the 872 participants who were HBsAg negative, 445 (51.0%) have received at least one dose of hepatitis B vaccine while 427 (49.0%) have not received any dose of hepatitis B vaccine.

**Table 3:** Participants in the study flow chart (*N* = 3132)

Variable:	Doctors (*N* = 297)		Nurses/Pharmacists/Lab workers (*N* = 716)		Admin staff (*N* = 2119)		Total (*N* = 3132)	
	*N*	%	*N*	%	*N*	%	*N*	%
Participants who knew their HBsAg status:								
Yes	162	54.6	282	39.4	449	21.2	893	28.5
No	135	45.4	434	60.6	1670	78.8	2239	71.5
Total participants	297	100.0	716	100.0	2119	100.0	3132	100.0
HBsAg of participants who knew their status:								
HBsAg Positive	1	0.6	6	2.1	14	3.3	21	2.4
HBsAg Negative	161	99.4	276	97.9	435	96.7	872	97.6
Total who knew their status	162	100.0	282	100.0	449	100.0	893	100.0
Participants who were HBsAg negative, and took/ did not take any dose of hepatitis B vaccine:								
Took at least a dose	98	60.9	155	56.2	192	44.1	445	51.0
Did not take any dose	63	39.1	121	43.8	243	55.9	427	49.0
Total who were HBsAg negative	161	100.0	276	100.0	435	100.0	872	100.0
Doses of vaccine taken by the participants:								
Participants who were HBsAg negative, and took one dose	8	8.2	26	16.8	122	63.5	156	35.1
Participants who were HBsAg negative, and took two doses	16	16.4	44	28.4	11	5.8	71	16.0
Participants who were HBsAg negative, and took three doses	74	75.4	85	54.8	59	30.7	218	48.9
Total who took at least a dose	98	100.0	155	100.0	192	100.0	445	100.0

### Reasons for non-uptake of hepatitis B vaccine by the participants

The reasons for non-uptake of hepatitis B vaccination by the 427 HBsAg negative participants included: cost of the vaccine (46/427, 10.8%), didn’t know where to receive the vaccine (203/427, 47.5%), didn’t believe they could be infected (28/427, 6.6%), and other reasons including the long vaccination schedule of the vaccine and lack of time (150/427, 35.1%). Details are as shown in Table 4.

**Table 4:** Reasons for non–uptake of Hepatitis B vaccine by the Participants (*N* = 427)

Reasons:	Doctors (*N* = 63)		Nurses/Pharmacists/Lab Workers (*N* = 121)		Admin staff (*N* = 243)		Total (*N* = 427)	
	*N*	%	*N*	%	*N*	%	*N*	%
Cost								
Yes	4	6.3	7	5.8	35	14.4	46	10.8
No	59	93.7	114	95.2	208	85.6	381	89.2
Don’t know where to take the vaccine								
Yes	34	54.0	35	28.9	134	55.1	203	47.5
No	29	46.0	86	71.1	109	45.9	224	52.5
Don’t believe I could be infected								
Yes	1	1.6	3	2.5	24	9.8	28	6.6
No	62	98.4	118	97.5	219	90.2	399	93.4
Others e.g. long vaccination schedule and lack of time								
Yes	3	4.8	19	15.7	128	52.7	150	35.1
No	60	95.2	102	84.3	115	47.3	277	64.9

### Factors associated with uptake of hepatitis B vaccination among participants that were hepatitis B negative

Table 5 shows factors associated with uptake of hepatitis B vaccination by the participants. After adjusting for age, sex, marital status, level of education, and professional categories, the Odds for uptake of hepatitis B vaccination were higher among single/separated/widowed participants compared to those that were married (AOR = 1.38, 95% CI = 1.01–1.92, *P* = 0.050). The Odds for uptake of hepatitis B vaccination were 22% lower among nurses compared to doctors (AOR = 0.78, 95% CI = 0.54–0.98, *P* = 0.037), while the Odds for the uptake of hepatitis B vaccination increased with increasing age (AOR = 1.30, 95% CI = 1.08–1.59, *P* < 0.001). The Odds for uptake of hepatitis B vaccination also increased with increasing duration of work in the hospital (AOR = 1.19, 95% CI = 1.09–1.32, *P* = 0.032). It was about 2.1 higher among participants who had secondary education compared with those that had primary education (AOR = 2.06, 95% CI = 0.75–5.61, *P* = 0.159), and about twice higher among those that had tertiary education than others that had less education (AOR = 1.96, 95 CI = 0.76–5.07, *P* = 0.164).

**Table 5:** Factors associated with Uptake of Hepatitis B Vaccination among participants that had Hepatitis B Negative (*N* = 872)

Variable:	Uptake of Hepatitis B Vaccination (*N* = 872)				Chi-square	*P* value	AOR (95% CI)	*P* value
	Yes	%	No	%	(X^2^)			
Sex								
Male	124	27.9	135	31.6	0.558	0.455	1.00	
Female	321	72.1	292	68.4			0.93 (0.69–1.24)	0.305
Age group (Years)								
< 30	60	13.5	80	18.7	14.048	0.003	1.00	
31–40	187	42.0	191	44.7			1.30 (1.08–1.59)	< 0.001
41–50	126	28.3	117	27.4			1.38 (1.09–1.65)	< 0.001
51–60	72 16.2		39	9.2			1.41 (1.12–1.73)	0.032
Marital status								
Married	327	73.5	293	68.6	1.981	0.576	1.00	
Single/Separated/Widowed	118	26.5	134	31.4			1.38 (1.01–1.92)	0.050
Education level								
Primary	8	1.8	13	3.0	1.825	0.768	1.00	
Secondary	58	13.0	56	13.2			2.06 (0.75–5.61)	0.159
Tertiary	379	85.2	358	83.8			1.96 (0.76–5.07)	0.164
Professional Categories								
Doctors	98	22.0	63	14.7	11.108	0.049	1.00	
Nurses/Pharmacists/Lab workers	155	34.8	121	28.4			0.78 (0.54–0.98)	0.037
Admin staff	192	43.2	243	56.9			0.23 (2.67–5.69)	0.241
Duration of work in the hospital								
< 1 Year	33	7.4	124	29.0	18.742	< 0.001	1.00	
1–5 Years	109	24.5	95	22.3			1.19 (1.09–1.32)	0.032
> 5–10 Years	128	28.8	93	21.8			1.26 (1.08–1.45)	0.043
> 10 Years	175	39.3	115	26.9			1.28 (1.12–1.49)	0.042

*AOR* Adjusted Odds Ratio, *95% CI* 95% Confidence Interval, *Reference category* 1, X^2^ = Chi-square

## Discussion

This study showed that the uptake of hepatitis B vaccination among HCWs in Enugu, Nigeria was poor. This is similar to the study conducted in Pakistan [15], but differs from other studies from India and Ethiopia [16, 17]. The authors found that 28.5% of the participants knew their hepatitis B status, and that 2.4% were hepatitis B positive, while 97.6% were hepatitis B negative. The observed low knowledge of hepatitis B status (28.5%) in this study could be due to absence of pre-employment screening for hepatitis B as well as lack of policy concerning hepatitis B screening in the facility. It also suggests that the facility has not been regularly conducting free or subsidized screening for its HCWs. While it is expected that all individuals who are hepatitis B negative should take the vaccination, while those who are hepatitis B positive receive treatment, the study found that only 51% of participants who are hepatitis B negative have received hepatitis B vaccination.

The overall uptake of hepatitis B vaccination among HCWs in this study was 14.2%. This is similar to what was observed in previous studies [18, 19]. However, it is lower than 22.4% reported in the similar study in 2006 [20], and also among HCWs in a teaching hospital in Ile-Ife, South-West Nigeria which revealed that 65% of the health workers have been vaccinated against hepatitis B virus [4]. The observed uptake in this study is also lower than 54.8% reported among theatre and laboratory workers at a teaching hospital in Imo state, Nigeria [21]. These differences could be due to the fact that the current study involved a larger population, and also administrative staff which were not involved in previous studies. This study also revealed that only 28.5% of the participants had prior knowledge of their hepatitis B status. This poor result could be due to the cost of screening for hepatitis B surface antigen and its poor accessibility in Nigeria. Regular free or subsidized screening programmes might bring improvement in this regard.

It was observed that a higher proportion of administrative staff have not received hepatitis B vaccination compared to nurses, doctors, pharmacists and laboratory technologists. This higher uptake of hepatitis B vaccination among the clinical than administrative staff could be as a result of their pre-employment training and education which might have included the importance and safety of vaccination to health. The management of health facilities should pay attention to administrative cadre of staff for improved uptake of hepatitis B vaccination among its HCWs. Similarly, the duration of HCWs that had worked in the facility also influenced the uptake of hepatitis B vaccination. Higher proportion of those who had worked for more than 5 years in the facility received hepatitis B vaccination than those who had worked for less. This result could be due to lack of policy concerning hepatitis B vaccination of workers in the hospital. Also higher proportion of participants older than 30 years of age received hepatitis B vaccination than those below 30 years old. This higher uptake of hepatitis B vaccination among older HCWs who are also more likely to have worked for longer duration might be due to their previous observations. It is possible they might have observed their colleagues suffer fulminant hepatitis and/or liver cancer as a result of possible non-uptake of hepatitis B vaccination. The reason for this higher uptake among this category of HCWs may also be due to previous hepatitis B vaccination related encouragements from colleagues. Such encouragement could lead to increase in awareness and knowledge of importance of screening and uptake of hepatitis B vaccination for those who are hepatitis B negative, and treatment for those who are hepatitis B positive. Formulation of policies to make screening for hepatitis B surface antigen and uptake of hepatitis B vaccination compulsory and at free or subsidized cost for all HCWs may bring improvement in uptake of hepatitis B vaccine.

This study found that 48.9% of those who were vaccinated had full coverage of the three doses of the vaccine, while 16 and 35.1% took two or one dose respectively. This is similar to the study conducted in Tanzania, and India among HCWs where 48.8 and 50% received three doses of the vaccine respectively [22, 23]. It is also similar to the study conducted among doctors and nurses in Lagos, Nigeria where 48.5% completed three doses of hepatitis B vaccination [24]. It is however lower than results documented in the study conducted among doctors and nurses in Iran which reported that 86.2% completed the recommended three doses of vaccine [25]. This difference might be due to the fact that the present study involved all health workers including administrative staff compared to study from Iran where only doctors and nurses were included. Thus, the differences in this study compared with other studies could be due to the inclusion of administrative staff, and the larger sample size. Interestingly, those who received three doses of vaccine in the current study is higher than the findings in other studies conducted in Nigeria, Sweden, Pakistan, and South Africa which reported 16.3, 29.7, 39.8, 37.2, and 19.9% respectively [20, 21, 26–28]. However, it is lower than the findings in a study done in Ethiopia where 61.2% of those vaccinated had received all 3 doses of the vaccine [17].

Our study revealed that age, staff category and duration of work in the facility significantly influenced uptake of hepatitis B vaccination, but no factor significantly influenced full vaccination status. However, after adjusting for confounders, the odds for full hepatitis B vaccination were higher among female participants than males (AOR = 1.17, 95%CI = 0.76–1.78, *P* = 0.265), tertiary education compared to primary education (AOR = 2.94, 95% CI = 0.64–12.43, *P* = 0.328), and among participants with longer duration of work (AOR = 1.23, 95%CI = 0.96–1.59, *P* = 0.106). These observations are similar to findings from some other studies which reported that sex, years of occupational practice, and educational status significantly influenced vaccination pattern [4, 20, 21].

The findings in the current study showed that 10.8% of the participants did not receive hepatitis B vaccination because of cost of vaccination, 47.5% did not know where to take the vaccination, 6.6% believed they could not be infected, while 51.1% gave other reasons such as long vaccination schedule, and lack of time. These findings are similar to a study conducted to find reasons for non-uptake of vaccine which reported inadequate vaccine information as a factor [11] and unavailability of vaccine and high cost of vaccine as major determinants [29]. The prevalence of HBV markers which includes individuals with HBsAg, anti-HBc, and anti-HBs who do not need hepatitis B vaccination is 72.5% in Nigeria [30]. Recommendations from American College of Physicians and the Centers for Disease Control and Prevention is that screening for HBV should include testing to three HBV screening sero-markers so that persons can be classified into the appropriate hepatitis B category and properly recommended to receive vaccination, counselling, and linkage to care and treatment [31]. However in Nigeria, accessibility and cost of hepatitis B serological tests for HBV markers is a great challenge. The authors’ opinion is that all HCWs should be screened for only HBsAg, and those that are negative should receive hepatitis B vaccination to reduce the cost and other challenges.

## Conclusions

This study revealed that uptake of hepatitis B vaccination as well as number of doses received was low among HCWs in Enugu, Nigeria. Age, staff category, and duration of work in the hospital, were independently associated with hepatitis B vaccination. It is therefore recommended that Management of health facilities in Nigeria should provide hepatitis B surface antigen screening facilities and hepatitis B vaccination sites for easy accessibility, and also subsidize the cost of screening for hepatitis B surface antigen and hepatitis B vaccination for all HCWs. They should also formulate policies that make screening for hepatitis B surface antigen and uptake of hepatitis B vaccination for hepatitis B negative HCWs compulsory and at free or subsidized cost. If possible, the Management should also frequently organize free screening for HBV for all its HCWs. This would increase the proportion of HCWs that know their hepatitis B status, as well as stimulate those that are hepatitis B negative and positive to receive the required vaccination and treatment respectively.

## Abbreviations

Anti-HBc: Antibody to hepatitis B core antigen
Anti-HBs: Antibody to hepatitis B surface antigen
AOR: Adjusted Odds Ratio
CI: Confidence interval
COR: Crude Odds Ratio
HBsAg: Hepatitis B surface antigen
HBV: Hepatitis B Virus
HCWS: Health Care Workers
OR: Odds Ratio
SPSS: Statistical Package for Social Sciences
UNTH: University of Nigeria Teaching Hospital
